# Population Frequency of *RYR1* and *CACNA1S* Variants Associated with Malignant Hyperthermia in the Korean General Population: A KOVA Database Analysis

**DOI:** 10.3390/genes17101176

**Published:** 2026-09-24

**Authors:** Jihyo Byon, Jimyeong Jeong

**Affiliations:** 1 College of Arts and Sciences, Saint Louis University, St. Louis, MO 63103, USA; lia.byon@slu.edu; 2 Department of Anesthesiology and Pain Medicine, Inha University College of Medicine, Incheon 22212, Republic of Korea

**Keywords:** malignant hyperthermia, *RYR1*, *CACNA1S*, Republic of Korea

## Abstract

**Background/Objectives:** Malignant hyperthermia (MH) is a rare but life-threatening pharmacogenetic disorder. It is most often caused by variants in *RYR1* and *CACNA1S*. The frequency of these variants differs across populations. However, no study has examined this in a general, unselected Korean population. **Methods:** We screened 81 *RYR1* and *CACNA1S* variants from the European Malignant Hyperthermia Group (EMHG) diagnostic panel (79 *RYR1*, 2 *CACNA1S*). We searched for these variants in the KOVA II database, which holds genetic data from 5305 healthy Koreans. Each variant was matched by exact genomic position, reference allele, and alternate allele. Korean allele frequencies for these four confirmed variants were then compared with the Genome Aggregation Database (gnomAD, v4.1.1) using Fisher’s exact test, and confirmed variants were mapped to established *RYR1* mutational hotspot regions. **Results:** Four *RYR1* variants were confirmed in the Korean cohort. This gave an overall carrier frequency of 0.075%, or about 1 in 1326 people. All four variants were classified as pathogenic or likely pathogenic in ClinVar/EMHG and all localized to *RYR1* mutational hotspots. Three of the four variants showed higher allele frequencies than gnomAD v4.1.1, ranging from 5.0-fold to 19.8-fold; the fourth, c.1565A>G, was not detected in gnomAD v4.1.1 and was significantly enriched relative to it (*p* = 0.0023). This variant was also reported as a hotspot in Taiwan. Neither of the two EMHG-listed *CACNA1S* variants (c.3257G>A, c.520C>T) was detected. **Conclusions:** This is the first population-level report of *RYR1* and *CACNA1S* variant frequency in Koreans. These findings reflect the carrier frequency of EMHG-listed variants rather than the clinical prevalence of MH susceptibility itself. All confirmed variants clustered in known mutational hotspots and were more common than in global databases. These findings provide a baseline reference that may help guide MH genetic testing in the Korean population.

## 1. Introduction

Malignant hyperthermia (MH) was first documented as an inherited familial disorder in the early 1960s, when a young Australian patient with a family history of anesthetic deaths developed a near-fatal hypermetabolic crisis during halothane anesthesia [1]. It is triggered by exposure to volatile anesthetics or the depolarizing muscle relaxant succinylcholine in susceptible individuals, producing uncontrolled myoplasmic calcium release, muscle rigidity, tachycardia, hypercapnia, and severe hyperthermia [2]. If not promptly recognized and treated, MH can progress to cardiac arrest and death; even with modern management, a North American MH registry analysis of 291 episodes reported a 2.7% cardiac arrest rate and a 1.4% mortality rate [3]. A nationwide clinical survey in Denmark estimated the incidence of fulminant MH at approximately 1 in 62,000 anesthetics when both a potent inhalation agent and succinylcholine were administered, while abortive or suspected MH occurred more frequently, in up to 1 in 4200 anesthetics under the same high-risk combination [4]. These findings underscore why early recognition and management of MH remain clinically important [3].

MH susceptibility is most commonly caused by variants in the ryanodine receptor 1 gene (*RYR1*), first linked to chromosome 19 through linkage analysis of susceptible families [5]; unselected exome sequencing later estimated pathogenic *RYR1* variants may occur in approximately 1 in 220 people (4 of 870 unselected exomes) in the general population, far exceeding the clinical incidence of overt MH, though one variant’s dominant-MHS pathogenicity was later disputed [6]. A smaller proportion of cases involve *CACNA1S*, encoding the skeletal muscle dihydropyridine receptor alpha-1 subunit, first identified via screening of a large MH pedigree [7]; subsequent exome studies identified novel pathogenic *RYR1* and *CACNA1S* variants in previously mutation-negative families, while also revealing extensive rare background variation in the general population, confirming that MH genetic architecture is highly heterogeneous [8]. This spectrum differs substantially between ethnic groups: a UK cohort study statistically reclassified 25 additional *RYR1* variants as likely pathogenic based on their significant enrichment relative to reference population databases, substantially expanding the diagnostic variant panel [9], a Singaporean whole-genome study found pathogenic *RYR1* frequencies varying significantly among Chinese, Malay, and Indian subgroups versus European databases [10], and panel testing of 247 Japanese MH families identified *RYR1*/*CACNA1S* variants at frequencies distinct from Western cohorts [11]. In Korea, prior work has been limited to case reports and small pedigrees, including one family with *RYR1* c.7304G>A (Arg2435His) and a seven-family screening identifying nine *RYR1* variants [12,13].

Despite this accumulating evidence of population-specific variation, no study to date has examined the frequency of MH-associated *RYR1* and *CACNA1S* variants in a general, unselected Korean population. Existing Korean data are derived exclusively from small numbers of clinically affected families rather than from population-representative sequencing, so it remains unknown which pathogenic and likely pathogenic variants are actually present among Koreans and at what frequency. Without this baseline information, clinicians cannot estimate how many otherwise healthy Koreans may carry an undiagnosed genetic susceptibility to MH, nor can genetic testing panels be confidently prioritized for variants most relevant to the Korean population. The present study therefore aims to characterize the spectrum and allele frequency of known pathogenic and likely pathogenic *RYR1* and *CACNA1S* variants in a large Korean general-population genome database and compare these findings to existing reference databases, providing the first population-level reference for MH genetic susceptibility in Koreans. Because systematic reference-database frequencies were not compiled for the full 81-variant panel, comparisons with gnomAD in this study were performed only for the variants confirmed present in the Korean cohort.

## 2. Materials and Methods

### 2.1. Data Source and Study Population

Variant data were obtained from the Korean Variant Archive (KOVA II) [14], a publicly accessible database maintained by the Korea Bioinformation Center (KOBIC) comprising combined whole-genome and whole-exome sequencing data (1896 whole-genome and 3409 whole-exome samples) from 5305 unrelated Korean individuals recruited across multiple Korean research institutions as a nationally supported reference database [14]. The cohort was unselected for MH-related phenotypes. Gene-specific variant tables for *RYR1* (chromosome 19) and *CACNA1S* (chromosome 1), including reference/alternate alleles, allele count (AC), allele number (AN), and allele frequency (AF), were retrieved through the KOVA web query interface using GRCh38 genomic coordinates. Records not annotated to *RYR1* or *CACNA1S* were excluded prior to analysis. Because sequencing depth varies by genomic position, AN reflects only alleles with sufficient coverage at each site and therefore varies across variants rather than being a fixed 2 × 5305 (10,610). Allele, carrier, and population frequency are used here in their standard, non-interchangeable senses.

### 2.2. Reference List of Pathogenic Variants

Known MH-associated variants were compiled from the European Malignant Hyperthermia Group (EMHG) online diagnostic mutation panel (https://www.emhg.org/, accessed on 8 August 2026, 09:22 KST), an actively curated resource built on the genetic and functional characterization framework described in the current EMHG guideline [2]. At the time of access, this panel comprised 79 *RYR1* and 2 *CACNA1S* variants meeting EMHG’s criteria for MH association; this figure reflects ongoing updates to the online panel and is larger than the 72 *RYR1* variants classified as pathogenic or likely pathogenic under the EMHG scoring matrix described in the current guideline [2]. For each variant, the corresponding GRCh38 genomic position, reference allele, and alternate allele were determined from ClinVar [15] using the MANE Select transcripts [16] NM_000540.3 (*RYR1*) and NM_000069.3 (*CACNA1S*). Pathogenicity classification (Pathogenic or Likely Pathogenic) for each variant followed American College of Medical Genetics and Genomics/Association for Molecular Pathology (ACMG/AMP) criteria [17] as recorded in ClinVar [15], which served as the primary reference for pathogenicity classification, consistent with prior population-based *RYR1* variant landscape studies [10]. These classifications were adopted from EMHG/ClinVar and not independently reassessed. The full list of 81 screened variants is provided in Supplementary Table S1.

### 2.3. Variant Matching and Statistical Comparison

Each EMHG-listed variant was cross-referenced against the KOVA dataset by exact match of genomic position, reference allele, and alternate allele; matches based on position alone were not considered sufficient, as multiple distinct substitutions can occur at the same nucleotide position. For each of the four variants confirmed present in KOVA, gnomAD allele count (AC) and allele number (AN) were retrieved individually via variant-specific lookup, rather than through a systematic bulk comparison of the full 81-variant panel; the Korean AC and AN were then compared against the corresponding values reported in the Genome Aggregation Database (gnomAD, v4.1.1) [18] using Fisher’s exact test (two-sided), implemented in Python (SciPy v1.x, Python 3.14.). Variants not found in gnomAD v4.1.1 were conservatively treated as zero-count (see Section Limitations). A *p* value < 0.05 was considered statistically significant; given the small number of observed carriers per variant, results were also interpreted in light of statistical power. The overall carrier frequency of pathogenic/likely pathogenic *RYR1* and *CACNA1S* variants among Koreans was estimated as the number of unique KOVA individuals carrying a matched variant divided by total cohort size.

### 2.4. Hotspot Mapping and Visualization

Confirmed variants were classified according to three *RYR1* mutational hotspot regions previously established in the literature: N-terminal (codons 34–614), central (codons 2163–2458), and C-terminal (codons 4136–4973) [10,19]. Corresponding exon boundaries for the N-terminal and central domains were determined by mapping these codon ranges onto the NM_000540.3 reference transcript. A linear protein schematic (Figure 1) illustrating these hotspot regions alongside the confirmed variant positions was generated using Python (Matplotlib v3.x).

## 3. Results

### 3.1. Overall Variant Spectrum

Screening of the EMHG diagnostic mutation panel (accessed 8 August 2026, 09:22 KST) [2] identified 81 candidate variants (79 in *RYR1*, 2 in *CACNA1S*) meeting EMHG’s criteria for MH association, of which ClinVar [15] classified 57 as Pathogenic and 24 as Likely Pathogenic per ACMG/AMP criteria [17]; the majority were missense substitutions. Cross-referencing this panel against the KOVA dataset by exact position and allele match confirmed the presence of four *RYR1* variants in the Korean population: c.1201C>T (p.Arg401Cys, Pathogenic), c.1565A>G (p.Tyr522Cys, Likely Pathogenic), c.1589G>A (p.Arg530His, Likely Pathogenic), and c.7282G>A (p.Ala2428Thr, Pathogenic). The two EMHG-listed *CACNA1S* variants, c.3257G>A and c.520C>T, were both absent from the KOVA cohort; no *CACNA1S* variant from the panel was identified in the Korean population.

### 3.2. Overall Population Carrier Frequency

There were 4 carriers of pathogenic or likely pathogenic *RYR1* variants among 5305 individuals in the KOVA cohort, corresponding to a carrier frequency of 0.075% (approximately 1 in 1326 individuals).

### 3.3. Comparison with gnomAD

Three of the four confirmed variants showed a numerically higher allele frequency in the Korean cohort than reported in gnomAD v4.1.1, ranging from 5.0-fold to 19.8-fold (Table 1); the fourth, c.1565A>G, was not detected in gnomAD v4.1.1. Only c.1565A>G reached statistical significance by Fisher’s exact test (*p* = 0.0023), reflecting its apparent absence from gnomAD despite being observed once among 3692 Korean alleles. The remaining three comparisons (c.1201C>T, *p* = 0.053; c.1589G>A, *p* = 0.183; c.7282G>A, *p* = 0.085) did not reach statistical significance, likely reflecting limited power given single-carrier observations.

### 3.4. Individual Variant Spotlight

The variant with the highest allele frequency among the four confirmed in this study, c.1565A>G (p.Tyr522Cys), was absent from gnomAD v4.1.1 despite being observed once among 3692 Korean alleles (Table 1).

### 3.5. Variant Location and Mutational Hotspot Analysis

All four confirmed variants localized to previously described *RYR1* mutational hotspot regions [10,19] (Figure 1): p.Arg401Cys, p.Tyr522Cys, and p.Arg530His fell within the N-terminal hotspot (codons 34–614, exons 2–19), while p.Ala2428Thr fell within the central hotspot region (codons 2163–2458, exons 39–47).

## 4. Discussion

This study characterized the spectrum and allele frequency of known pathogenic and likely pathogenic *RYR1* and *CACNA1S* variants in a general, unselected Korean population using the KOVA II database. The EMHG diagnostic mutation panel was cross-referenced against 5305 Korean genomes, using exact position and allele matching. This identified four *RYR1* variants present in the Korean population, corresponding to an overall carrier frequency of 0.075%. To our knowledge, this is the first study to report the population-level frequency of MH-associated genetic variants among Koreans unselected for MH-related phenotypes.

Other studies have also estimated how common these MH-related variants are, with mixed results. Our carrier frequency of 0.075% (about 1 in 1326 people) is close to the 0.125% reported in an unselected Singaporean cohort [10] and to a global estimate of 0.087% (about 1 in 1149) based on gnomAD allele frequencies [20]. In contrast, a different prior study reported a much higher frequency of about 1 in 220 people [6]. That estimate came from only 870 exomes with just 4 carriers, half sharing a since-disputed variant, so it rests on a much smaller sample and may be less precise. Overall, our finding agrees closely with other large, population-based studies, while smaller studies show wider variation.

All four confirmed *RYR1* variants localized to established mutational hotspot regions [10,19], which correspond to functionally critical domains of the RyR1 channel [21]. Three of the four also showed consistently higher allele frequencies than gnomAD v4.1.1 [18], ranging from 5.0-fold to 19.8-fold. This mirrors the Singaporean whole-genome study, where pathogenic *RYR1* variants were similarly hotspot-concentrated and more frequent in Asians than globally [10]. This quantification, based on population-representative sequencing rather than case series, extends the earlier Korean case reports [12,13].

The variant with the highest frequency among the four confirmed in this study, c.1565A>G (p.Tyr522Cys), was also the only comparison reaching statistical significance against gnomAD v4.1.1 (*p* = 0.0023), reflecting its apparent absence from that database. Treating this absence as a zero-count is a conservative approximation, not evidence of a true global frequency of zero, given the limited East Asian representation in gnomAD; this finding is therefore best regarded as a preliminary signal rather than confirmatory evidence. No correction for multiple testing was applied across the four comparisons, as these were independent tests of pre-specified variants rather than an exploratory scan, consistent with comparable population-genetics studies [10,22,23]. This variant has independently been reported as a potential hotspot in a Taiwanese MH cohort [20]. Its convergent detection across two East Asian populations suggests regional enrichment rather than a Korea-specific finding, consistent with the ethnicity-specific enrichment pattern previously observed for *RYR1* c.7268T>A among Singaporean Indians [10].

*CACNA1S* encodes the skeletal muscle dihydropyridine receptor alpha-1 subunit, which couples membrane depolarization to *RYR1*-mediated calcium release. The R1086H mutation disrupts this coupling’s normal negative regulatory influence, enhancing RyR1 sensitivity to both voltage and caffeine activation [24]. In contrast, neither EMHG-listed *CACNA1S* variant was identified in KOVA (c.3257G>A; c.520C>T). This negative result warrants caution, as only two *CACNA1S* variants are currently EMHG-listed, giving limited power to detect true absence. *RYR1* and *CACNA1S* may therefore not share the same East Asian enrichment pattern, underscoring the need for larger or independent Korean cohorts to clarify this issue.

Together, these findings suggest that Korean MH genetic testing panels may benefit from prioritizing *RYR1* mutational hotspots, and c.1565A>G in particular, rather than relying solely on variant lists derived mainly from European cohorts [9]. Genetic testing is recommended by EMHG guidelines for individuals with a family history of MH, personal adverse anesthetic reactions, or exertional rhabdomyolysis and can serve as a primary investigation for index cases in select circumstances [2]. No dedicated Korean-population reference frequencies for these variants previously existed; the present findings therefore provide a baseline that may assist clinicians in interpreting incidental findings of these variants in otherwise healthy individuals undergoing unrelated genetic testing.

This study leveraged KOVA II, a national reference dataset that addresses the underrepresentation of Korean and other non-Caucasian populations in variant databases and has previously confirmed that Koreans form a genetically distinct population [14]. Because participants were unselected for MH phenotypes, KOVA is well suited to estimating baseline carrier frequency rather than penetrance. This design parallels the Singaporean SG10K cohort [10] and Gonsalves et al.’s exome-based study [6]. A key methodological strength of this study was verifying variants by exact position and allele match, which was necessary since several EMHG positions overlapped with distinct, non-pathogenic Korean substitutions.

### Limitations

This study used aggregate, anonymized allele frequency data from KOVA without access to individual clinical histories; it was therefore not possible to determine whether carriers displayed any MH-related phenotype, or to estimate variant penetrance. For the same reason, individual-level genotype data were unavailable, so zygosity and potential overlap among the four carriers could not be confirmed beyond noting that each variant was observed as a single allele (AC = 1). Second, only variants included in the EMHG diagnostic mutation panel were screened; novel or non-listed pathogenic *RYR1*/*CACNA1S* variants present in the Korean population would not have been captured by this approach. Third, each confirmed variant had only a single carrier, so the resulting estimates carry wide uncertainty, and three of four comparisons against gnomAD did not reach statistical significance; these findings should therefore be treated as preliminary rather than confirmed. This reflects a structural constraint rather than a design flaw: because MH itself is rare, its causal variants are expected to occur at correspondingly low frequencies in any general population, so even a national-scale dataset such as KOVA is likely underpowered to detect statistically robust differences for individual variants, and substantially larger cohorts will be needed to confirm these frequency estimates. Fourth, despite the substantially larger sample size of gnomAD v4.1.1 relative to earlier releases, East Asian ancestry remains underrepresented relative to European ancestries in that database, which could inflate apparent Korean-specific enrichment for variants that are simply underreported globally [10]. Undetected sample overlap between KOVA and gnomAD’s East Asian group cannot be excluded, though this would bias comparisons toward the null, making it unlikely to explain the significant enrichment observed for c.1565A>G.

## 5. Conclusions

This study characterizes *RYR1* and *CACNA1S* variant frequency among unselected Koreans, identifying four *RYR1* variants at a carrier frequency of 0.075%, while no *CACNA1S* variant was detected. This reflects variant carrier frequency rather than the clinical prevalence of MH susceptibility itself. All confirmed variants localized to established mutational hotspot regions and occurred at higher frequencies than in global databases, consistent with patterns seen in other Asian populations. The convergent detection of c.1565A>G in both Korean and Taiwanese cohorts suggests possible enrichment shared with at least one neighboring population, warranting confirmation in additional East Asian cohorts. These findings establish a baseline reference for MH genetic testing in Koreans.

## Figures and Tables

**Figure 1:**
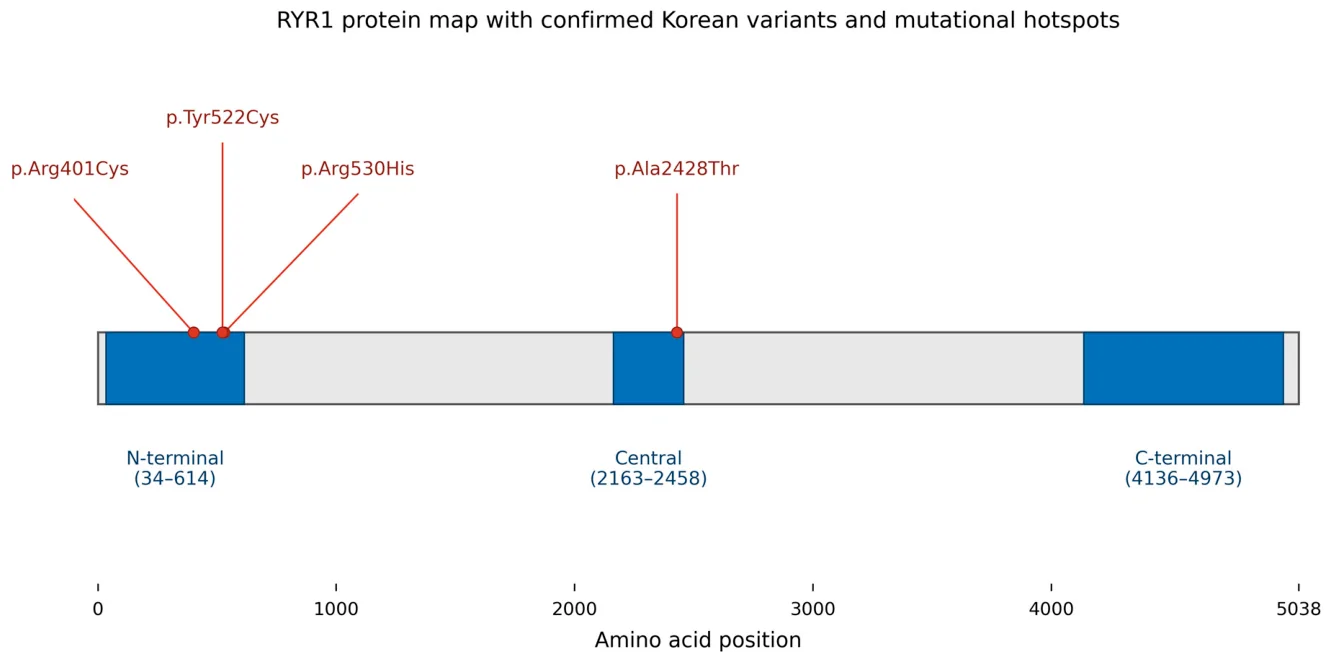
*RYR1* protein map showing the four Korean-confirmed variants relative to previously established mutational hotspot regions. Linear schematic of the *RYR1* protein (5038 amino acids) with an amino acid position axis. Blue segments indicate the three previously established mutational hotspot regions: N-terminal (codons 34–614), central (codons 2163–2458), and C-terminal (codons 4136–4973) [9,19]. Red markers indicate the positions of the four *RYR1* variants confirmed in the Korean KOVA cohort in this study. p.Arg401Cys, p.Tyr522Cys, and p.Arg530His cluster within the N-terminal hotspot; p.Ala2428Thr falls within the central hotspot. No confirmed variant localized to the C-terminal hotspot.

**Table 1:** Comparison of Korean (KOVA) and gnomAD (v4.1.1) allele frequencies for confirmed *RYR1* variants.

Variant (HGVS)	Protein Change	Classification	Korean AF (AC/AN)	gnomAD AF (AC/AN)	Fold Difference	Fisher’s Exact *p*
c.1201C>T	p.Arg401Cys	Pathogenic	0.000147 (1/6806)	0.0000074 (12/1,613,996)	19.8×	0.053
c.1565A>G	p.Tyr522Cys	Likely Pathogenic	0.000271 (1/3692)	Not detected	—	**0.0023**
c.1589G>A	p.Arg530His	Likely Pathogenic	0.000147 (1/6814)	0.0000291 (47/1,613,974)	5.0×	0.183
c.7282G>A	p.Ala2428Thr	Pathogenic	0.000147 (1/6814)	0.0000124 (20/1,613,982)	11.8×	0.085

Note: Allele frequencies (AFs), allele counts (ACs), and allele numbers (ANs) for the four RYR1 variants confirmed by exact position and allele match in the KOVA II Korean cohort (*n* = 5305), compared with gnomAD (v4.1.1) [18]. Fold difference was calculated as Korean AF divided by gnomAD AF. Statistical comparison used Fisher’s exact test (two-sided); c.1565A>G, which was not detected in gnomAD v4.1.1, was conservatively treated as zero-count. “—” indicates the variant was not detected in gnomAD, so no fold difference could be calculated. Bold *p* value indicates statistical significance (*p* < 0.05).

## Data Availability

The variant frequency data analyzed in this study are available from the Korean Variant Archive (KOVA II) at the Korea Bioinformation Center (KOBIC) query interface. Restrictions apply to the availability of individual-level data; aggregate allele frequency data used in this study can be retrieved by querying *RYR1* and *CACNA1S* at the KOVA web portal. gnomAD (v4.1.1) allele frequency data used for comparison are publicly available at https://gnomad.broadinstitute.org.

## Supplementary Materials

The following supporting information can be downloaded at https://www.mdpi.com/article/10.3390/genes17101176/s1, Table S1: The 81 EMHG-listed RYR1 and CACNA1S variants included in the diagnostic panel screened in this study.
